# Challenges and recommendations for mental health providers during the COVID-19 pandemic: the experience of China’s First University-based mental health team

**DOI:** 10.1186/s12992-020-00591-2

**Published:** 2020-07-09

**Authors:** Shitao Chen, Feihan Li, Chaihua Lin, Yuge Han, Xilun Nie, Robert N. Portnoy, Zhihong Qiao

**Affiliations:** Faculty of Psychology, Beijing Normal University, Beijing, 100875 China

**Keywords:** COVID-19, Mental health provider, Challenges, Recommendations, China

## Abstract

Coronavirus Disease is impacting the entire world. As the first country that has needed to confront this disease, China has responded with unprecedented and hugely successful public health initiatives. Almost simultaneous with the awareness of the potential for widespread loss of life, the first Chinese university recognizing the likely psychological impacts of COVID-19, assembled the first university-based professional team to offer pandemic-related mental health services to the Chinese public. This paper describes the work that we provided and the challenges encountered. The challenges are described in four contexts: the organizational/systemic level, the technical perspective, the therapeutic process, and the ethical aspects. We also provide recommendations on what we can do in the short term, and future improvements that can be made.

## Background

The outbreak of the Coronavirus Disease 2019 (COVID-19) has created global health concerns and devastated the entire world. As the first country impacted by COVID-19, China experienced different stages of this epidemic (later, pandemic) from late 2019 to June 2020 [1], but now appears to have the incidence of daily new confirmed cases under control [2]. This overwhelming public health emergency was shown to have a substantial negative impact on people’s mental health, leading to clinical and sub-clinical disorders, such as anxiety, depression, acute stress disorder, Post-traumatic stress disorder (PTSD) and other mental health symptoms [3, 4], as well as to the exacerbation of pre-existing mental health conditions. Similar impacts were found by Qiu’s team, who conducted the first nationwide, large-scale survey of psychological distress of the Chinese people during the COVID-19 epidemic [1]. To prevent people from being psychologically overwhelmed during this tumultuous period, the staff psychologists at Beijing Normal University (BNU) quickly met and reached a consensus about what was needed. We consequently formed the first university-based psychological team in China, making available free mental health services through telecommunication to all of the Chinese people [5]. Our target population included everyone who needed psychological first aid, but the main areas of focus were on those vulnerable individuals who were suffering from the disease and their family members, front-line medical staff, and people whose relatives had died as a result of this disease [5].

With a sense of urgency, our team discussed the appropriate scope of our services and the structure of our work in a single day, and then completed recruitment and pre-implementation training over 2 days. The recruiting procedure included applicants filling out a screening form listing relevant clinical experience and also providing a phone number for a professional reference. The management team then scrutinized the applicants’ clinical backgrounds, contacted references, and then selected mental health professionals who met our internally established criteria. Among these recruited volunteers, roughly half were master’s-level students who had received their training from the Clinical and Counseling Psychology program at BNU with more than 300 clinical hours, and the other half were either alumni of BNU with specific counseling qualifications and crisis intervention experience, or counselors who were trained overseas and received their mental health licensure from abroad.

**Fig. 1:**
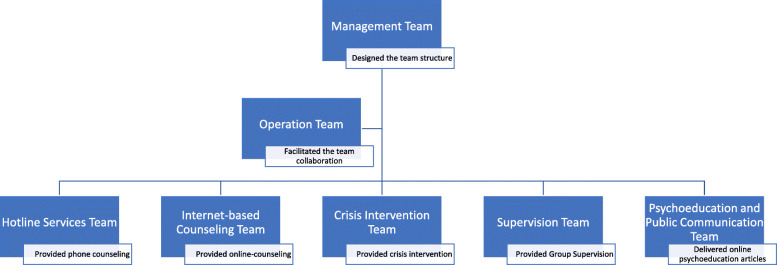
Team Structure

**Fig. 2:**
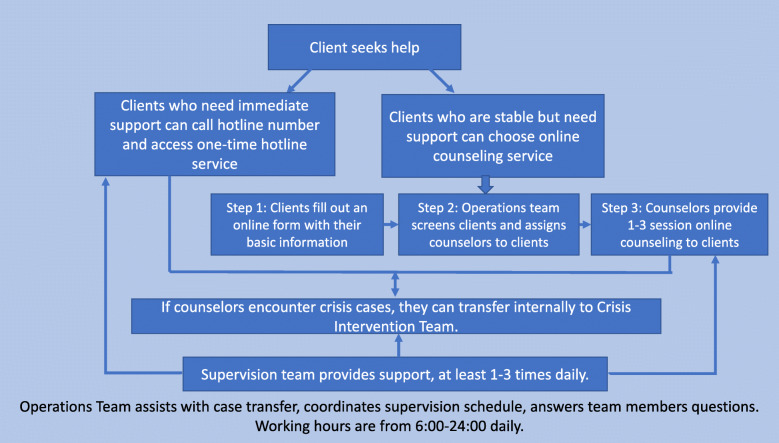
Working Procedure

The overarching coordinating team collaborated online and was composed of 6 different smaller teams: the Hotline Services team, the Internet-based Counseling team, the Crisis Intervention team, the Supervision team, the Psychoeducation and Public Communications team, and the Operations team. All counselors also received daily regular supervision to discuss their cases and to process their emotional reactions. The leader of our supervision team also organized regular trainings for counselors to ensure their competence level in dealing with this unique public health emergency. Figure 1 represents the team structure, and Fig. 2 demonstrates the working procedure.

As of the time of this writing, our team has provided mental health services to over 7000 people, and engaged in crisis intervention for 157 people. The supervision team has offered more than 150 sessions of group supervision and provided 21 workshops to counselors. We also wrote 56 psychoeducational articles, reaching over 2 million readers. To understand the help-seekers’ needs and oversee the quality of our services, we required all counselors to document their cases and submit a short summary through an online survey system on a daily basis. The online survey system asked the counselors to identify the client’s gender, age range, location, the category of their identity (e.g., first-line medical staff member, COVID-19 patient, family member of a patient), the category of the problem they were facing (e.g., worry about being infected, grief and loss, family conflict), their key emotional symptoms (e.g., anxiety, depression, anger, PTSD symptoms), and whether they need to be transferred to the crisis intervention team, as well as the intervention already delivered. To ensure the confidentiality of clients’ personal information, we confirmed with the online survey company that all information would be kept confidential before we agreed to utilize their services. In addition, all notes can only be accessed by the program supervisors, and no client’s identifiable information was submitted online. Periodically, we scrutinized the clinical notes and summarized the trend of needs of our clients. Based on our findings, our team published a book [6], in which we introduced some common forms of psychological distress that people have faced during this emergency, and made recommendations to different populations, including the general population, frontline medical staff, individuals who might be infected, individuals who are confirmed to have this disease, elderly people, children and adolescents, and people who are dealing with loss and grief. Our work was highly recognized and valued by the Chinese government. The Ministry of Education in China promoted our work and recommended that the education system in China should follow our team’s lead and provide hotline and online counseling services to people who are dealing with distress during this crisis period [7, 8]. Consistent with this recommendation, as of April, 2020, China has developed over 600 hotlines and online counseling services offered to the Chinese people [7, 8].

### Challenges we encountered

As the first university-based mental health program in China to build a working team and provide hotline and online counseling services, perhaps not surprisingly, there were a number of lessons learned during this process. The challenges and related lessons learned are described in four contexts: the organizational/systemic level, the technical perspective, the therapeutic process, and the ethical aspects.

#### Organizational/systemic level challenges

Although the current pandemic shares some common features with the Severe Acute Respiratory Syndrome (SARS) pandemic in 2003, the differences between them brought new challenges for providers of interventions on the related mental health issues. On the national level, a comprehensive crisis prevention (primary prevention) and intervention (secondary and tertiary intervention) system had not been previously established [1]. We had to explore, debate, and then establish our own working procedures with little experience to guide us from previous public health crises. The challenges we experienced involved multiple legal, professional, and technical issues.

##### Legal and ethical dilemmas

The first challenge was the lack of clear legal regulation regarding the scope of services, which increased the risk of becoming involved in legal and ethical dilemmas. The Mental Health Law of the People’s Republic of China is the only law that regulates mental health practice in China, and it endeavors to differentiate “psychological counseling” and “psychotherapy” [9]. The former refers to interventions conducted by counselors with individuals who typically have relatively minor or more developmental issues, while the latter refers to interventions conducted by medical professionals in a medical facility with individuals who have formal psychiatric diagnoses. Unfortunately, as referenced in the “Translator’s notes” in the English version of the law [10], nowhere is there a definition of what types of treatment are considered psychotherapy, or of what type of training is necessary to be qualified to provide psychotherapy. Nevertheless, the law’s intent is clear on the following points:

Psychological counselors shall not provide psychotherapy and shall not diagnose or treat mental disorders. (Mental Health Law of the People’s Republic of China; Chapter II, Article 23).

Further, it is explicitly stated that:

“When psychological counselors detect possible mental disorders in clients, they shall advise them to seek treatment at a medical facility that meets the criteria for treating mental disorders specified in this law.” (Mental Health Law of the People’s Republic of China; Chapter II, Article 23).

Consequently, even though the law attempts by exclusion to differentiate the scope of services that a counselor can and cannot provide, when callers initiated contact with our counselors, we were unable to consistently ensure that those who sought our services did not potentially have disorders that were diagnosable, had not previously been diagnosed, or, more generally, were not currently struggling with more severe mental health disorders. In fact, during our work, we encountered a significant number of help-seekers who, it emerged, did have or likely met criteria for diagnoses of severe mental disorders. Such needs for service during this national and international crisis required timely, but difficult, referrals to psychiatric facilities. At the same time, however, we recognized that we had to provide initial counseling interventions that we determined were within the legal scope of our services in this public health emergency or risk denying essential and otherwise unavailable mental health services to the most needy of our citizens in their time of crisis. Furthermore, to deny services in the midst of an emergency would not only have been unacceptably cold and cruel, but contravenes one of the central dictates of the helping professions: “First, do no harm”. Through a close reading of the relevant mental health law, we determined that we could, in fact, maintain a strict adherence to the law by limiting our interventions to counseling and, when required, crisis intervention, and still have a beneficial impact on both the individual and societal levels. While this required us to address any potential confusion about these issues in our trainings, we nevertheless found that we were able to come to a reasonable interpretation of a set of laws that was not designed for and could not have anticipated such extreme circumstances.

For example, during the operation of our hotline service, we came across a regular caller who appeared to have some psychotic symptoms and who announced that he was planning to hurt some security guard in his community. The counselor on one hand, listened patiently on the phone and provided emotional support while collecting information and, on the other hand, quickly sought support from the supervisors and also consulted one of the psychiatrists on staff. The supervisors collected information from the counselor, and assisted her to evaluate the severity of the case. Based on the information we received, the team made a decision to contact the police, as well as one of the caller’s family members, and made appropriate recommendations and referrals to the family member.

##### Professional qualification dilemmas

The second systemic challenge was related to the lack of standards for the regulation of professional qualifications. Since the cancellation of the counselors’ qualification exam in 2017, there has been no licensure that would standardize the qualifications of mental health professionals in China counselor [11]. Therefore, being a Counseling or Clinical Psychologist certified by the Chinese Psychological Society (CPS) is the only organizational approach that allows one to be considered a qualified mental health professional. When coupled with the lack of systematic clinical training in China in general, it was not an easy matter for us to assess applicants’ professional competence during the recruitment phase. In order to protect the quality of services, we therefore had to start small by primarily recruiting counselors who had received, at a minimum, systematic master’s-level training from BNU, with more than 300 h of direct service and over 100 h of supervision, or counselors who were trained abroad with similar requirements. Then, we expanded the recruitment to counselors referred by our alumni. We asked all the applicants to report their training background, including degrees, names of training programs, direct clinical hours, hours of receiving individual and group supervision, and experience in crisis intervention, and to provide one reference. While the desire of Chinese counselors to contribute was greatly appreciated, we ended up screening out approximately 61.5% of the applicants. All of the accepted counselors had a minimum of a master’s-level degree in Counseling or Counseling Psychology, with a mean number of clinical hours of 1153 and individual supervision hours of 140, as well as group supervision hours of 190. In addition, we provided intensive online group supervision to all counselors throughout the project, but especially during the first 4 weeks of service, not only to support the counselors, but also to monitor their performance through case discussion, role play, and other supervision strategies. We were able to identify problematic counseling behaviorss through supervision. For example, we communicated our concern and subsequently prohibited a therapist from providing services due to their lack of maintaining appropriate boundaries in hotline counseling. We also had to ask counselors to discontinue the provision of services when we detected that their personal issues were being triggered and interfered with their professional work.

Similar to the challenges in assessing counselors’ competence or possible impairment, it was difficult to recruit supervisors and assess their level of competence. We therefore only recruited experienced supervisors with whom we were already familiar. Such a recruitment strategy was able to provide reassurance regarding supervisors’ qualifications and competence, but it also limited the pool of supervisors. Due to the lack of standards in professional qualifications and regulations for supervisors, we only felt confident in collaborating with a very small number of supervisors, which was deemed necessary in order to lower the risk of unacceptably diminishing the quality of our services. A breakdown of our counselors and supervisors’ backgrounds and clinical experience is presented in Table 1.

**Table 1:** Background information of Volunteer Counselors and Supervisors

		Hotline Team *N* = 120	Internet Based Team *N* = 104	Crisis Intervention Team *N* = 24	Supervision Team *N* = 48
Gender	Male	18	15	4	12
	Female	102	89	20	36
Education Level	Bachelor	6	0	0	4
	Master in training	4	44	0	0
	Master	94	59	16	18
	Doctoral in training	2	0	0	2
	Doctoral	14	1	8	24
Clinical Hours	100–200	0	56	0	0
	200–500	35	34	4	0
	500–1000	36	5	11	0
	> 1000	49	9	9	48

##### Crisis intervention dilemmas

Another significant systemic challenge was related to working with clients who were in imminent danger. All counselors recruited had been formally trained in suicide assessment and developing a safety plan, and had at least one course in crisis intervention. For the ones who participated in the hotline service or the crisis team, they additionally had to have some practical experience in dealing with crisis or suicide intervention. Identifying a person (typically a relative or friend) to serve as the client’s emergency contact is a widely accepted practice in regular counseling services. Usually, this information is collected before the first session. However, as is the case with almost all crisis intervention, when a client calls the hotline for help, the therapist does not have the opportunity to collect emergency contact information before the phone call. The therapist therefore needs to collect information from the client in crisis on the phone, which often is not an easy process. Given the nature of crises, the client’s sense of urgency makes it much less likely that they will be able to tolerate this sort of questioning [12]. When a hotline emergency occurs, a police or medical response is sometimes needed. This presented another challenge. The knowledge of the location of the client is critical, but the policy of employing mobile phone technology to ascertain the location of an individual might vary by country. In China, only in criminal cases can one get permission to use location technology. For mental health emergencies, a policeman usually goes to the listed home address when searching for a person who is posing a serious and imminent risk of suicide.

For example, there was one time our team received a phone call in the middle of the night, in which the help-seeker announced that she planned to kill herself in a few hours. We searched the caller’s location through the calling system, but later realized that the location only identifies the caller’s phone registration area, not her actual location at that moment. The hotline counselor was not able to get information regarding where she was right then, nor could the counselor get the caller’s name, personal id number, or emergency contact information. When the counselor sought guidance from the supervision team, the supervisor quickly called the local police emergency number and reported the imminent danger presented by the caller and consulted regarding how best to collaborate. The police collected as much information as possible to evaluate the emergency level, and the counselor at the same time continued talking to the caller, attempting to calm her and keep her on the phone until further help could be provided. Because the exact location of the caller was hard to identify, the police department had to collaborate internally, first to identity in which area the caller was located and then to allocate resources to implement the plan to rescue the caller, all of which took an understandably, but excruciatingly long time. Luckily, the counselor was finally able to calm that caller down and the caller agreed not to attempt suicide, and, in the end, the police no longer needed to search for the caller. This lesson taught us that hotline counselors should have knowledge about the national policy regarding the use of mobile phone location technology and the process of working collaboratively and effectively with law enforcement personnel, in case they find themselves intervening with a client in a potentially lethal crisis. We also recommend hotline counselors not to work alone; in case of emergency, they need to have some support already in place, so that the support system can help to contact other law enforcement or the hospital system staff.

#### Technical level challenges

##### Technical difficulties with cellphones

The hotline services also encountered a few technical challenges. Due to the quarantine policy, counselors had to use their own cellphones, and work from home. The hotline system was designed to assign phone calls randomly to counselors’ cell phones. As a result, counselors’ cell phone numbers are only protected when receiving phone calls. If they spoke with a client in crisis and needed to call back, the callers would be able to see their personal phone numbers. To solve the problem, we employed a second hotline system provided by another telephone company, which can block counselors’ phone numbers under all circumstances. Additionally, it quickly became apparent that the telephone company would not always have enough workers or programmers to repair any technical failures that occurred during the quarantine. This led to some communication interruptions. Counselors on the hotline services team therefore had to inform callers at the beginning of every call that communication interruptions happen occasionally, so, should this occur, the callers would need to call back to continue the services. The person who receives the phone call, however, might be a different therapist. The caller can then decide whether to work with the new therapist or not. This was far from ideal. We found that communication interruption had some, perhaps obvious, negative influences on the therapeutic relationship. Both counselors and callers felt frustrated when they couldn’t get in touch after the interruption. Having to repeat their presenting problem was both tiring and irritating to some callers.

##### Difficulties with accurate workload documentation

Working-at-home brought further difficulties related to documenting the overall workload. Some phone calls to the hotline were inappropriate for our counseling services, such as a request for a media interview, misdialing, or misunderstanding the purpose of the hotline. The resulting data generated by the hotline system consequently did not accurately reflect the real work of counselors. The same problem occurred with online counseling. To remediate these statistical errors, we chose to count the counselors’ work based upon the clinical notes that they generated.

Online supervision shared the same difficulties, but it also had its own unique challenges due to employing new technology. We used online video and audio conference software, like ZOOM or ZHUMU, to provide group supervision every day. Since ZOOM and ZHUMU do not have the function of providing statistics, we had to count the supervision hours manually. Moreover, the level and quality of engagement during the online supervision was difficult to ascertain. Initially, some supervisees chose to turn off the video camera to improve internet stability during supervision, but it was then difficult to distinguish a silent participant from someone who just turned off the video, and may have been doing something else, with only audio playing in the background. We therefore established a policy that required video, in order to address these concerns. Supervisees had to turn on the camera in the supervision in order to claim their hours. If a supervisee’s internet could not support video conferencing for the entire time, she/he had to inform the supervisor before the supervision session, and turn on the video at the beginning and toward the end, in order for the supervision hour to be counted.

##### Social justice challenges

The challenges we ran into in the systemic, resource, and technical aspects of our work highlighted for all of us of the issues related to social justice. According to our data through April 8th, 2020, 4.67% of hotline callers were under the age of 18, and 0.78% above 69; 10.54% of online counseling clients were under the age of 18, and only one client was above 69-years-old, comprising 0.58% of the total online counseling population. Access to and familiarity with computers, smart phones and the internet reflect privilege, both financially and educationally. Such privilege might also differentially impact an individual’s comprehension of mental health issues and related help-seeking behaviors. In addition, the availability of medical and referral resources differs significantly by region. When making referrals, we often ran into challenges in finding local referral resources. Help-seekers’ financial situation also limited their ability to access referral resources, even when they could be identified.

#### Clinical challenges

##### Practical dilemmas

Clinically, we also faced challenges that are unique to this pandemic. A lot of help-seekers expressed practical dilemmas and a sense of helplessness due to the lockdown of public facilities and the dearth of resources. For instance, with the rapid increase of the rate of infection and the inadequacy of medical supplies (e.g., testing kits, hospital beds, medicines) at the outset of the pandemic, people were worried that they could not get the treatment they needed, especially the ones who were already experiencing symptoms. Patients with chronic illness who needed regular hospital visits could not get physical treatment in a timely fashion. With the COVID-19 infection spreading globally at this stage, some Chinese students and other Chinese nationals overseas who had made the decision to fly back were not able to do so due to the high cancellation rate of international flights. According to our clinical records, among the 150 callers from overseas, 30% of them felt isolated in their current situation, and 19% of them were worried about the frequently changing international regulations and policies regarding the pandemic. While working with individuals who were facing these practical dilemmas, our counselors consistently felt and expressed a strong desire to support them, but experienced a sense of helplessness at the same time. This trend was initially the anecdotal impression of the supervisors, but it was subsequently strongly supported by a review of the supervisory records. Consequently, supervisors were also found to have spent a substantial amount of supervisory time on their supervisees’ countertransference reactions. To support counselors when they had a sense of helplessness, most of the supervisors created a safe space during group supervision, and helped the counselors to process their feelings; supervisors not only acknowledged the difficult time we were all facing and therefore normalized their sense of frustration and helplessness, but also identified the appropriate and helpful counseling skills that the counselors had already utilized in supporting these callers. The supervisors also shared other techniques and resources that the counselors could use, and employed some relaxation exercises to help the counselors to increase their self-compassion and decrease their mental distress.

##### Dilemmas with providing services in severe cases

Another therapeutic challenge relates to the adjustment and limitation of using tele-media as a way to conduct counseling. During service provision, we encountered several suicidal calls and repeat callers who had severe mental health issues (e.g., bipolar disorder, clients with psychotic symptoms, and clients with long-term addiction issues). For counselors who were not specifically trained to deal with severe clients in a one session context, and who were also new to the hotline/online type of service, a lot of them felt a sense of incompetence, and worried that they were not effectively helping their clients. For example, 70% of the time in supervision, counselors were expressing doubts about their therapy skills (e.g., “Is it right if I respond in this way?”, “What else can I do to help?”, and “How should I respond when my client expresses very intense emotion”). We also fielded questions about how to get an accurate evaluation of clients’ current mental health status and form a comprehensive case conceptualization with limited nonverbal information and having such a brief time working with clients. For repeat callers, especially the ones who might have personality disorders or who were experiencing a manic episode, the call usually stirred up the emotions of the entire team. There was an inherent tension between providing sufficient support to these special help-seekers, while simultaneously protecting the emotional well-being of our counselors, including the need to address the potential for burnout.

The issue of protecting against counselor burnout emerged as a central concern in the therapeutic process. Research suggests that, after 3–6 h of uninterrupted work, a significant proportion of helpers report impaired functioning associated with elevated symptoms of psychological distress [13]. Large-scale surveys also suggest that helpers who experience elevated personal symptoms of psychological distress are unable to communicate or use their core skills effectively [14, 15]. These results are consistent with what we observed during our work on this project. We noticed that after receiving 3 to 4 intense calls over a 3-h shift, our counselors started to experience emotional exhaustion, and expressed more uncertainty about the interventions they provided. Not only did the intensive work in a single shift impact the counselors’ sense of well-being, the uncertainty of the overall duration of our work, the unpredictable amount of need, and the increased severity of the callers’ issues, all led to burnout and fatigue on our team, and made the work more difficult.

#### Ethical challenges

##### Challenges working with media

In the course of developing and delivering mental health services during the pandemic, we were also faced with several unique ethical challenges. The first challenge concerned issues regarding public media, which can be seen as both a blessing and a curse. As our service received more public attention, many from the media contacted our team for various interview requests. We considered this a great opportunity to increase public awareness of mental health issues related to COVID-19. However, when dealing with the media, many of them were overly interested in obtaining “in-depth” interviews with counselors that asked them to provide caller stories. Some even went so far as requesting us to film our counseling and supervision process. This revealed the public’s (and the media’s) general lack of awareness of the ethical boundaries regarding privacy and confidentiality issues within mental health services. In this process, our team had to repeatedly explain and emphasize the boundaries of privacy and confidentiality. We value the great opportunity to collaborate with the media to educate the public, but it was a struggle to find a balance between responding to the media’s wants and needs and protecting our clients by upholding important ethical standards. According to the Ethical Principles of Psychologists and Code of Conduct (“APA Ethics Code”) (American Psychological Association, 2017, [16]), ethical standard 4.07 makes it clear that psychologists do not disclose client information in public media unless “they take reasonable steps to disguise the person or organization,” and “the person or organization has consented in writing.” The team initially considered adding an item to the online informed consent form regarding whether or not the client would be willing to share their story. However, considering the vulnerable position clients are in when they seek help, this was viewed as potentially exploitative. Furthermore, because clients do not have the ability to view the final media product before it goes to the public, they would not have the chance to withdraw their consent if they were uncomfortable with it. Given these considerations, we had to turn down all the story-sharing requests, and ultimately felt confident that this was the right call. Despite this, some media tried to approach some of the counselors privately to solicit stories, and we had to make clear and repeated announcements to prohibit our volunteers and counselors from agreeing to such unethical interview requests.

##### Challenges with telepsychology

The second ethical challenge concerned the practice of telepsychology. Due to the nature of our hotline service, we could not obtain written informed consent signed by both the counselor and the client. Furthermore, due to the lack of legal regulations, there is no equivalent in Chinese law to the Health Insurance Portability and Accountability Act (HIPAA), and therefore no platform has been designed to provide an encrypted communication environment for counselors and clients. This presents great threats to the security and transmission of client data with online counseling services, but, given the urgency of the circumstances and the implications for the Chinese public, and after considerable internal debate, we decided that we could not afford to allow this limitation to shut down our much-needed efforts.

### Recommendations

#### Short-term recommendations

Based on the challenges we experienced, we wish to share several recommendations that can be considered for mental health practitioners or agencies who decide to start similar volunteer intervention programs within the context of the current or future pandemics.

First, we recommend setting clear standards for counselor and supervisor qualifications. The following were the standards we used to select counselors: 1) minimum of a master’s degree (or master in training) in clinical/counseling psychology or psychiatry; 2) minimum of 300 h of direct service for hotline counselors, and minimum of 100 h of direct service for internet-based counselor; 3) previous crisis intervention training and experience; and 4) having received a minimum of 50 h of individual and group supervision. Additionally, the standards we used to select supervisors were: 1) minimum of a master’s degree in Clinical/Counseling Psychology or Psychiatry; 2) minimum of 1000 h of direct service; 3) systematic supervision training and past experience in providing supervision; and 4) registered counselor or supervisor with the Chinese Psychological Society or a Level II certified counselor with the Ministry of Human Resources and Social Security.

Second, we would like to stress the importance of clarifying the boundary regarding the nature and scope of one’s mental health services. To begin with, our services were set up to help support people who were distressed by COVID-19. Therefore, it was important to make appropriate referrals when calls came in for other issues. In addition, it was essential to clarify the short-term nature of both our hot-line and online counseling services, and provide guidelines to counselors on how to handle frequent callers, especially those with severe mental health concerns. During our project, we held at least 10 unique group supervision sessions, where we gathered counselors who received calls from the same caller. By gathering information from all the counselors involved in such cases, we were able to form comprehensive case conceptualizations of the particular callers, and then provide targeted interventions and, when appropriate, referrals to them. Furthermore, although crisis intervention might have been beyond the initial scope of our planned services, it was inevitable and unavoidable that we would receive crisis calls, so it was also important to have a crisis intervention plan in place [12].

Third, efforts must be made to avoid counselor burn-out. The initial workload of our mental health service was enormous. Some counselors were receiving phone-calls non-stop during their 3-h shifts. Exposure to others’ distress put some counselors at risk for vicarious traumatization. These, combined with counselors’ own feelings of anxiety and helplessness in the face of COVID-19, greatly increased the risk of burn-out. As such, it is critical to set limits on the number of shifts a counselor takes per week, particularly the number of night shifts, as many crisis calls occur during the night. To prevent counselor burn-out, timely supervisory support is also critical. Besides the daily supervision sessions, we had on-call supervisors in place to provide support to counselors as needed. In addition, we held several workshops on the topic of self-care to further raise counselor awareness of the importance of this issue.

Fourth, we learned how important it was to accept our limits as mental health providers. During our service provision, we dealt with many practical concerns in the lives of the callers, such as shortages of supplies, environmental constraints, potential psychiatric diagnoses, and economic problems. These are problems that cannot be resolved through counseling, and it is not within our scope of care to even try to resolve these problems for the callers. It is important to remind our counselors that our primary role is to provide a safe space to contain the callers’ distress, to listen and provide support, to help the callers to draw upon their resources and strengths to better manage these difficulties, and to provide referrals to other services when appropriate [17]. By accepting our own limits and the limitations of the hotline service, we can protect our counselors from the constant threat of feeling ineffective or helpless.

Fifth, we learned through difficult experience that we had to be firm and assertive with the media. When dealing with the understandably curious media, but especially with those who were overly interested in probing for “juicy” stories, it was extremely important to patiently and nondefensively explain the boundaries of confidentiality and firmly assert what we could and could not share. It was also important to get the entire team on the same page about media communication and interviews. We also recommend having a trusted person designated as the media spokesperson to respond to all interview requests, and firmly communicating the need for us to review for accuracy and approve the final product before it goes to the public.

Last but not least, in order to avoid problems related to the technology, it is important to communicate with clients about the risks and limitations of telepsychology. Though it is difficult to obtain a written informed consent with hotline counseling, it is important to ensure that all conversations start with an oral informed consent. If there is no platform that could provide an encrypted communication environment, we must make sure to communicate the potential risk to the clients and take whatever steps possible to maximize the protection. For example, if possible, one should set a passcode for video rooms and only give access to the room to clients, and avoid leaving any written communications, to ensure the confidentiality of the conversation.

#### Long-term recommendations

The great demand for mental health services during the current pandemic has demonstrated that the public increasingly recognizes the importance of their own mental health and of mental health intervention. The opposite side of challenge is opportunity. The current pandemic has highlighted some of our limitations and specifically provides a crucial opportunity to improve the regulation of the counseling profession in China. In order to facilitate the development of the counseling profession, it is necessary to have licensure and regulation of professional qualifications and the scope of practice in place. A comprehensive primary prevention and secondary and tertiary intervention system for future public health crises, as well as related postvention plans, are also needed. With a sound overarching system in place, the delivery of mental health services in public health crises in the future would likely unfold much more smoothly.

As the public has now recognized the importance of recognizing and addressing mental health concerns, it is a good time for mental health professionals to promote psychoeducation. The utilization of social media and collaboration with professional media organizations can be helpful in terms of eliminating, or at least mitigating, the stigma related to emotional problems, mental illness, and help-seeking. Developing individualized media campaigns can meet the different needs of the diverse population (e.g., creating short videos on social media to reach adolescents and adults, or making TV programs for the elderly population who did not grow up with the newer technologies). The development of new technology has served to broaden the Chinese people’s understanding of the scope and content of mental health services, thereby providing new opportunities for educating the public.

It is also important to consider new ethical challenges that might come with the utilization of new technologies. The Chinese Psychological Society’s Ethics Code (2nd edition) [18] has published rules for online professional services and media collaboration. Counselors need to be aware of the risks involved in dealing with the issues of confidentiality, informed consent for services, crisis intervention, and publicity through the various media, when providing services over the internet.

## Conclusions

The initiative, attention to detail, and perseverance required for the work that we undertook was challenging, but rewarding. As mental health professionals, we are proud that we were able to work together in a collaborative fashion and use our psychological knowledge to assist the people of China through this pandemic. We are also aware that the limitations that we experienced have motivated us to continue to work on improving the mental health system and its regulation in China, growing our professional competence in working with a wider variety of clients through different platforms, and enhancing our capacity for navigating difficult ethical boundaries. Even though the work we have presented in this article only reflects the practices in China, as well as for our work with Chinese citizens abroad, we hope that our experiences can elicit further thought and reflection about the specific societal, cultural, and governmental influences we all face in providing mental health interventions during COVID-19 and future global crises. As the entire world must come together in the face of this devastating pandemic, we have a newfound appreciation for our global interdependence and therefore recognize how important it is that we mental health professionals share our knowledge and experience, thereby assisting in the development of effective and efficient mental health practices throughout the world.

## Data Availability

Not applicable.
